# Examining the Influence on Perceptions of Endometriosis via Analysis of Social Media Posts: Cross-sectional Study

**DOI:** 10.2196/31135

**Published:** 2022-03-18

**Authors:** Julian Matthias Metzler, Dimitrios Rafail Kalaitzopoulos, Laurin Burla, Gabriel Schaer, Patrick Imesch

**Affiliations:** 1 Department of Gynecology University Hospital Zurich Zurich Switzerland

**Keywords:** endometriosis, social media, Facebook, Instagram, influencer, engagement

## Abstract

**Background:**

Social media platforms, such as Facebook and Instagram, are increasingly being used to share health-related information by “influencers,” regular users, and institutions alike. While patients may benefit in various ways from these interactions, little is known about the types of endometriosis-related information published on social media. As digital opinion leaders influence the perceptions of their followers, physicians need to be aware about ideas and beliefs that are available online, in order to address possible misconceptions and provide optimal patient care.

**Objective:**

The aim of this study was to identify and analyze frequent endometriosis-related discussion topics on social media in order to offer caregivers insight into commonly discussed subject matter and aspects.

**Methods:**

We performed a systematic search using predefined parameters. Using the term “endometriosis” in Facebook’s search function and a social media search engine, a list of Facebook pages was generated. A list of Instagram accounts was generated using the terms “endometriosis” and “endo” in Instagram’s search function. Pages and accounts in English with 5000 or more followers or likes were included. Nonpublic, unrelated, or inactive pages and accounts were excluded. For each account, the most recent 10 posts were identified and categorized by two independent examiners using qualitative content analysis. User engagement was calculated using the numbers of interactions (ie, shares, likes, and comments) for each post, stratified by the number of followers.

**Results:**

A total of 39 Facebook pages and 43 Instagram accounts with approximately 1.4 million followers were identified. Hospitals and medical centers made up 15% (6/39) of the Facebook pages and 5% (2/43) of the Instagram accounts. Top accounts had up to 111,600 (Facebook) and 41,400 (Instagram) followers. A total of 820 posts were analyzed. On Facebook, most posts were categorized as “awareness” (101/390, 25.9% of posts), “education and research” (71/390, 18.2%), and “promotion” (64/390, 16.4%). On Instagram, the top categories were “inspiration and support” (120/430, 27.9% of posts), “awareness” (72/430, 16.7%), and “personal story” (72/430, 16.7%). The frequency of most categories differed significantly between platforms. User engagement was higher on Instagram than on Facebook (3.20% vs 0.97% of followers per post). On Instagram, the highest percentage of users engaged with posts categorized as “humor” (mean 4.19%, SD 4.53%), “personal story” (mean 3.02%, SD 4.95%), and “inspiration and support” (mean 2.83%, SD 3.08%). On Facebook, posts in the categories “awareness” (mean 2.05%, SD 15.56%), “humor” (mean 0.91%, SD 1.07%), and “inspiration and support” (mean 0.56%, SD 1.37%) induced the most user engagement. Posts made by hospitals and medical centers generated higher user engagement than posts by regular accounts on Facebook (mean 1.44%, SD 1.11% vs mean 0.88%, SD 2.71% of followers per post) and Instagram (mean 3.33%, SD 1.21% vs mean 3.19%, SD 2.52% of followers per post).

**Conclusions:**

Facebook and Instagram are widely used to share endometriosis-related information among a large number of users. Most posts offer inspiration or support, spread awareness about the disease, or cover personal issues. Followers mostly engage with posts with a humoristic, supportive, and awareness-generating nature. Health care providers should be aware about the topics discussed online, as this may lead to an increased understanding of the needs and demands of digitally proficient patients with endometriosis.

## Introduction

Endometriosis, defined as the occurrence of endometrium-like tissue outside the uterus, is a chronic, incurable disease affecting about 10% of women [1]. Main symptoms include dysmenorrhea, dyspareunia, chronic pelvic pain, and subfertility. Although it may lead to severe and sustained restrictions in women’s private, social, and professional lives, both men and women have little knowledge about the condition [2], and even general practitioners’ expertise about the disease is scarce [3]. This dilemma is further aggravated by the high prevalence of dysmenorrhea in adolescents, leading to a trivialization of pelvic pain. These factors mutually reinforce delayed diagnosis, withheld care, and lack of awareness. As women are dissatisfied with the public and medical aid they receive [4,5], it is understandable that they turn to other forms of support.

The internet has become the primary source for health information for people all over the world [6]. In addition to static websites, social media plays an increasing role as a channel for medical information [5]. Platforms such as Facebook and Instagram are used by the majority of US adults [7], and the number of health-focused social media accounts has been skyrocketing, with daily growth rates of up to 28% in 2020 [8]. Patients with endometriosis may benefit from this development in various ways. The process of informing oneself about health topics on social media platforms is private, yet personally tailored, allowing for sharing of personal anecdotes and interactions with others, if desired. Thereby, social and emotional support may be received [5,9]. Support in the form of disease management tips, experiences, and mental health support are valuable subjects among young adults with chronic disease as well as baby boomers and older adults [10,11].

On social media, content can be created by, and shared among, regular users, commercial companies, or nonprofit organizations alike. While any user can share personal or general health-related posts with their friends or with the public via their personal stream, other forms of information sharing have emerged in order to focus on specific topics. Two frequently observed variants are the creation of topic-specific Facebook pages and Instagram accounts. This allows for the promotion and distribution of topic-specific content and the dedicated recruitment of followers.

Regarding endometriosis, several hundred disease-related Facebook pages have been created so far [6]. Recently, “influencers” have increasingly gained importance on most social media channels, blurring the line between user-generated and advertorial content. It has been shown that these digital opinion leaders are able to change the attitudes of their followers, increase acceptance of the information provided, and even influence the intention to buy corresponding products [12].

Even though there is an abundance of endometriosis-related information online, filtering and assessing the quality of information can be challenging, as people make little use of source credibility [13]. Available information on endometriosis is often of low quality, inaccurate, or skewed toward the diagnosis or it conveys negative connotations, while high-quality information is challenging for a lay audience to comprehend [14,15]. These circumstances can induce feelings of fear and helplessness [16].

Nevertheless, internet health information seeking has become increasingly popular, and a growing number of patients with endometriosis can be expected to have acquired considerable knowledge on their condition before a consultation. While this may be challenging at first, it can improve the patient-physician relationship when the patient is able to discuss the information with the physician [17]. Naturally, this requires health care professionals to have an overview of the topics at hand.

While several studies have performed social media content analysis for different gynecological conditions on Instagram and Facebook [18-21], only few focused on endometriosis [5,22,23], and information about popular topics is scarce. Due to this paucity of data, it is unclear which topics prevail on both Instagram and Facebook and whether user engagement differs between platforms.

In this paper, we analyzed the nature of posts shared on endometriosis-related public Facebook pages and Instagram accounts and compared them between platforms, granting an additional tool to improve counseling, address possible misconceptions, and fulfill the patient’s expectations to be more engaged in health-related decision-making.

## Methods

### Design

We performed a cross-sectional analysis of public, endometriosis-related social media posts on Facebook and Instagram between August 3 and 5, 2020. The timing was selected with sufficient time distance from known events such as Endometriosis Awareness Month in March, which could have acted as a confounder. Facebook and Instagram were chosen as data sources for their high popularity [7]. The search was restricted to results in English and those publicly accessible to all Facebook and Instagram users.

### Data Selection

In order to identify relevant Facebook pages, we performed a search using the term “endometriosis” in Facebook’s search function on August 3, 2020. We deliberately included only Facebook pages, as Facebook groups are often private and require approval by a moderator to gain access.

Additionally, we performed a search on BuzzSumo, a website offering tools and search utilities for content discovery, content research, and identification of influencers across different social media platforms. In contrast to regular popularity ranking by likes, this website has the capability of ranking relevant Facebook pages by additional metrics such as “engagement,” an integrative score including reactions, comments, shares, and likes [24]. Results were structured by relevance and by likes in order to maximize the results.

Of all search results, the *information section* was screened together with the last 10 public posts to assess the scope and aim of each page. Pages were considered relevant when they covered endometriosis as their main topic. When endometriosis was not the main topic (eg, pages focusing on general health or nutrition), a minimum of 10 endometriosis-related posts within the last 2 years were considered necessary to qualify. Relevant pages in English with 5000 or more likes were included for further analysis. This cutoff was defined in order to limit analysis to content with a considerable reach and influence. Pages (ie, accounts) that did not meet the inclusion criteria (ie, endometriosis not the main topic, <10 endometriosis-related posts within 48 months, or <5000 followers) were excluded.

On Instagram, we searched for relevant accounts using the search terms “endometriosis” and “endo.” The search was conducted on August 5, 2020. Follower lists of relevant accounts were also screened in order to maximize search results. Account names and number of followers were extracted into a Microsoft Excel spreadsheet and sorted by number of followers. All relevant accounts in English with 5000 or more followers were included.

Of all eligible Facebook pages and Instagram accounts, the 10 most recent wall posts in Facebook or the 10 most recent posts in Instagram were analyzed further.

### Content Analysis, Codebook, and Categories

A systematic qualitative content analysis approach was used, as this is a standard process in interactive media content analysis [25,26]. A codebook was developed in order to systematically analyze the content of all relevant posts and to file them into predefined groups. The codebook contained 10 categories or groups together with a short explanation about each category’s applicability. For example, the “inspiration and support” category was defined as “all posts containing tips, support (mental, moral, etc), and inspirational texts, quotes, and memes.” The 10 content categories were defined as follows: “education and research,” “awareness and outreach,” “nutrition, food, and diet,” “sport and lifestyle,” “inspiration and support,” “personal story,” “patient requests,” “scientific inquiries,” “humor,” and “promotion of product or service.” An 11th category (ie, “other”) was available if none of these 10 categories seemed suitable. The categories were selected based on a previously published study on social media usage [19]; they were adjusted in order to fit the formulated research questions and to be more aligned with the investigated social media services, since the cited study was performed on Twitter and not Facebook or Instagram. Therefore, the categories “nutrition, food, and diet,” “patient requests,” “scientific inquiries,” and “sport and lifestyle” were introduced to test their occurrence in the data sample, while the categories “celebrity story,” “political,” and “news” were omitted.

Of all included Facebook pages and Instagram accounts, the 10 most recent endometriosis-related wall posts on Facebook or the 10 most recent posts on Instagram, as of August 5, 2020, were analyzed according to the codebook. Each post was coded as a single topic by two independent coders. Coders received an introduction to the codebook and a training set of 10 posts. If results differed between coders, the content was examined by a third person, and each case was discussed until agreement could be reached. Facebook pages and Instagram accounts were classified as health care professionals or medical centers if (1) their bio or page information section declared this classification or (2) if the content of their posts made this classification explicit. This categorization was conducted by a single reviewer (Reviewer A).

### Calculations and Metrics: User Engagement

To measure the impact of posts and the amount of interactions the content earned relative to reach, different metrics were calculated [27]. Total engagement was defined as the sum of all user interactions with a post. Likes, comments, and shares (Facebook only) were added together with equal weighting, as follows:

Total engagement per post = likes × 1 + comments × 1 + shares × 1

As an example, a post with 100 likes, 20 comments, and 10 shares would result in a total engagement of 130. Next, the percentage of account followers engaging with that post was obtained by calculating the engagement rate by reach (ERR) using the following formula:

ERR = total engagement per post / total followers × 100

Additionally, we calculated the percentage of followers liking, commenting, or sharing a post.

### Statistical Analysis: Interobserver Reliability

Statistical analyses were performed with SPSS Statistics for Windows (version 27; IBM Corp). Data were presented as numbers and percentages. For categorical data, chi-square tests and analyses of variance were used. A *P* value below .05 was considered statistically significant.

Chance-adjusted measurement of interobserver reliability was calculated using the Randolph free-marginal multi-rater κ [28], with κ values less than 0.40 considered as “poor,” values from 0.40 to 0.75 considered as “intermediate to good,” and values above 0.75 considered as “excellent.”

### Ethical Considerations

According to Swiss law, this is not a research project under the Swiss Human Research Act (Humanforschungsgesetz) and, therefore, no authorization is required.

## Results

### Pages, Accounts, and Posts

A total of 39 Facebook pages and 43 Instagram accounts were identified, with approximately 1.4 million followers. Top accounts had up to 111,600 (Facebook) and 41,400 (Instagram) followers, with a median follower number for Facebook and Instagram of 11,800 (IQR 7450-19,750) and 10,200 (IQR 7420-16,900), respectively. Hospitals and medical centers made up 15% (6/39) of the Facebook pages and 5% (2/43) of the Instagram accounts. A total of 820 posts were included for categorization: 390 (47.6%) Facebook posts and 430 (52.4%) Instagram posts. Interrater agreement was 62.8% (245/390) for Facebook, with a free-marginal κ of 0.59 (95% CI 0.54-0.64), showing substantial agreement. For Instagram posts, the interrater agreement was 57.9% (249/430), with a κ of 0.54 (95% CI 0.49-0.59).

### Top Categories by Frequency

All pages (ie, accounts) covered more than one topic (ie, published posts in several categories). There was a significant difference in topics between platforms, as shown by categorization frequency. On Facebook, most posts were categorized as “awareness” (101/390, 25.9% of posts), “education and research” (71/390, 18.2%), and “promotion of product and service” (64/390, 16.4%), with only a few posts addressing “sport and lifestyle” (6/390, 1.5%). As a benchmark, only 1.8% (7/390) of posts were categorized as “other.”

On Instagram, the top categories were “inspiration and support” (120/430, 27.9% of posts), “awareness” (72/430, 16.7%), and “personal story” (72/430, 16.7%). Only 0.2% (1/430) of posts were patients asking specific health questions (ie, “patient requests”) or calls for scientific study participation (ie, “scientific inquiries”). A total of 6.7% (29/430) of posts were categorized as “other.”

Out of 11 categories, 9 (82%) differed significantly in the frequency of their occurrence on Facebook and Instagram, suggesting that different topics were posted depending on the platform. Posts concerning “nutrition, food, and diet” and “sport and lifestyle” were published on both platforms without a significant difference (Table 1).

**Table 1:** Frequencies of post categories on Instagram as compared to Facebook.

Category	Facebook					Instagram				*P* value
	Posts (n=390), n (%)	ERR^a^ (likes), %^b^	ERR (comments), %^c^	ERR (shares)%^d^	Total ERR, %	Posts (n=430), n (%)	ERR (likes), %^b^	ERR (comments), %^c^	Total ERR, %	
Awareness	101 (25.9)	0.93	0.28	0.84	2.05	72 (16.7)	2.60	0.13	2.73	.001
Education and research	71 (18.2)	0.20	0.07	0.12	0.39	31 (7.2)	2.20	0.14	2.34	<.001
Promotion	64 (16.4)	0.27	0.03	0.03	0.33	49 (11.4)	0.68	0.06	0.74	.04
Inspiration and support	54 (13.8)	0.38	0.05	0.13	0.56	120 (27.9)	2.72	0.11	2.83	.001
Personal story	46 (11.8)	0.26	0.05	0.09	0.40	72 (16.7)	2.79	0.23	3.02	.02
Nutrition, food, and diet	14 (3.6)	0.08	0.15	0.02	0.25	12 (2.8)	1.53	0.15	1.68	.51
Patient requests	11 (2.8)	0.12	0.12	0.01	0.25	1 (0.2)	1.16	0.95	2.11	.002
Scientific inquiries	8 (2.1)	0.08	0.02	0.01	0.11	1 (0.2)	2.00	0.17	2.17	.01
Humor	8 (2.1)	0.54	0.11	0.26	0.91	36 (8.4)	4.04	0.15	4.19	<.001
Other	7 (1.8)	0.16	0.04	0.02	0.22	29 (6.7)	2.32	0.23	2.55	<.001
Sport and lifestyle	6 (1.5)	0.33	0.06	0.05	0.44	7 (1.6)	0.38	0.08	0.46	.92
All categories^e^	390 (100)	0.30	0.09	0.14	0.54	430 (100)	2.04	0.22	2.26	N/A^f^

^a^ERR: engagement rate by reach; ERR = total engagement per post / total followers × 100.

^b^The percentage of followers liking a post from a given category.

^c^The percentage of followers commenting on a post from a given category.

^d^The percentage of followers sharing a post from a given category.

^e^ERR values in this row are mean values of the 11 rows above.

^f^N/A: not applicable; *P* values in this column compare the category frequency between Facebook and Instagram; since the final row includes frequencies of 100% for both platforms, a *P* value was not calculated.

When combining both platforms, the top three categories were “inspiration and support” (174/820, 21.2% of all analyzed posts), “awareness” (173/820, 21.1%), and “personal story” (118/820, 14.4%). Infrequent categories included “sport and lifestyle” (13/820, 1.6%), “patient requests” (12/820, 1.5%), and “scientific requests” (9/820, 1.1%). Table 2 shows the combined frequency of all posts on Instagram and Facebook.

**Table 2:** Combined frequency of categories on Instagram and Facebook.

Category	Posts (N=820), n (%)
Inspiration and support	174 (21.2)
Awareness	173 (21.1)
Personal story	118 (14.4)
Promotion	113 (13.8)
Education and research	102 (12.4)
Humor	44 (5.4)
Other	36 (4.4)
Nutrition, food, and diet	26 (3.2)
Sport and lifestyle	13 (1.6)
Patient requests	12 (1.5)
Scientific inquiries	9 (1.1)

### User Engagement

User engagement was assessed based on 430 Instagram posts from 43 accounts (563,500 total followers) and 390 Facebook posts from 39 pages (831,900 total followers).

On both platforms, posts were typically liked, shared, or commented on by less than 5% of the followers, with a higher percentage of users engaging on Instagram than on Facebook. Across all categories, followers were over 3 times as likely to share or like a post on Instagram as compared to Facebook. On average, 3.20% (SD 3.65%) of followers engaged with a post on Instagram (liking a post: mean 3.01%, SD 3.41%; commenting a post: mean 0.19%, SD 0.37%) as compared to 0.97% (SD 8.02%) on Facebook (liking a post: mean 0.53%, SD 3.18%; commenting a post: mean 0.12%, SD 1.23%; sharing a post: mean 0.31%, SD 3.65%). Posts made by hospitals and medical centers generated higher user engagement than posts by regular accounts on Facebook (mean 1.44%, SD 1.11% vs mean 0.88%, SD 2.71% of followers per post), and Instagram (mean 3.33%, SD 1.21% vs mean 3.19%, SD 2.52% of followers per post).

### Top Categories by Engagement

#### Facebook

On Facebook, the top three categories by engagement were “awareness,” “humor,” and “inspiration and support.” Posts in these categories induced 2.05% (SD 15.56%), 0.91% (SD 1.07%), and 0.56% (SD 1.37%) of users to engage with that post, in contrast to only 0.11% (SD 0.13%) of followers engaging with calls to participate in scientific studies (ie, “scientific inquiries”). Of note, certain categories, such as “humor” or “sport and lifestyle,” accounted for a low number of posts, yet generated a lot of engagement (Table 1).

Next, we compared the number of Facebook posts in each category with the engagement rate of each category. A weak correlation was found between category frequency and ERR (*R*^2^=39.2%), showing that posts that induced high engagement tended to be posted more often or vice versa. Posts addressing disease awareness not only made up for 25.9% (101/390) of posts but also generated over 3.8 times more engagement than the average posts, performing at 382% of the expected engagement rate. Table 3 shows the engagement-inducing performance of posts for all categories on Facebook.

**Table 3:** Performance of Facebook posts by category.

Category		Proportion of mean performance (engagement), %^a^
**Overperforming posts**		
	Awareness	382
	Humor	169
	Inspiration and support	104
**Underperforming posts**		
	Sport and lifestyle	81
	Personal story	75
	Education and research	73
	Promotion	61
	Nutrition, food, and diet	46
	Patient requests	46
	Other	43
	Scientific inquiries	20

^a^100% represents the expected mean engagement across all categories.

#### Instagram

On Instagram, the top categories were “humor,” “personal story,” and “inspiration and support,” inducing mean user engagement of 4.19% (SD 4.53%), 3.02% (SD 4.95%), and 2.83% (SD 3.08%) of followers, respectively. Interestingly, “sport and lifestyle” scored last, with a mean user engagement of 0.46% (SD 0.64%) of followers (Table 1). Of note, the mean user engagement across all categories (0.54%, SD 0.52% on Facebook; 2.26%, SD 0.99% on Instagram) differed from the ERR described in the User Engagement section, as the latter was weighted for post frequency. As noted before, categories generating higher engagement tended to be posted more frequently or vice versa, leading to a higher ERR in pages and accounts that pursued this strategy. For Instagram, this correlation was weaker than for Facebook (*R*^2^=14% vs 39%). For example, even though humorous posts induced the most engagement on Instagram, they only accounted for 8.4% (36/430) of all posts. Table 4 shows the engagement-inducing performance of posts for all categories on Instagram.

**Table 4:** Performance of Instagram posts by category.

Category		Proportion of mean performance (engagement), %^a^
**Overperforming posts**		
	Humor	185
	Personal story	134
	Inspiration and support	125
	Awareness	121
	Other	113
	Education and research	104
**Underperforming posts**		
	Scientific inquiries	96
	Patient requests	94
	Nutrition, food, and diet	75
	Promotion	33
	Sport and lifestyle	20

^a^100% represents the expected mean engagement across all categories.

## Discussion

### Principal Findings

In this paper, we were able to quantify Facebook’s and Instagram’s popularity with regard to endometriosis. Over 80 pages and accounts with approximately 1.4 million followers were included. By analyzing 820 posts, we were able to specify the diversity of endometriosis-related content that was shared and discussed among Instagram and Facebook users.

Facebook’s top three categories—“awareness,” “education and research,” and “promotion”—may be explained by the intended use of the Facebook pages. Similar to a personal profile, a page enables organizations and other entities to create a public presence, which is visible to everyone on the internet [29]; this makes it a viable tool to generate topic-specific awareness, broadcast educational content, and promote products or services.

Our results are mostly in line with what was recently reported by Towne et al [5]. Even though a direct comparison is limited due to partly different categories, we classified a similar percentage of Facebook posts as “research and education” (18.2%) in our study compared to “education” in Towne et al (21%), and we found a low percentage of posts in the categories “nutrition, food, and diet” (3.6%) in our study compared to “recipe” in Towne et al (0.83%), “humor” in our study (2.1%) compared to “humor” in Towne et al (4.70%), “scientific inquiries” in our study (2.1%) compared to “survey” in Towne et al (0.34%), and “other” in our study (1.8%) compared to “other” in Towne et al (1.10%).

Similarly, Wilson et al [23] found a high percentage of posts on their endometriosis-related Facebook page to have informational content.

As described, certain categories were found to be less common than others; for example, only a few posts contained disease-specific questions from users posed to their followers (“patient requests”: Facebook 2.8% of posts; Instagram 0.2% of posts). A possible explanation is that such intimate questions tended to be asked in closed groups and forums or via direct messages rather than publicly. Furthermore, some categories may be underrepresented because of the topics being discussed on different pages: an account’s main topic may differ depending on the number of followers. For every account with over 5000 followers, there were numerous accounts with 50 to 500 followers, highlighting that information is spread not only from the top down, but also between smaller groups of peers. Without being fully investigated in this study, these smaller Facebook pages often tended to focus on the personal history or story of a single patient. This may explain a relative underrepresentation of the category “personal story” in the study results.

This is the first study showing that content creators share different topics depending on the platform, as popularity and frequency of topics vary between Facebook and Instagram. While most posts on Facebook were creating educational or promotional awareness, on Instagram, the topics were found to be somewhat more “intimate,” with a focus on inspiration and support, awareness, and personal stories. This interpretation may be valid for different reproductive topics as well. When analyzing fertility-related accounts on Instagram, Blakemore et al [19] found a high percentage of posts containing inspiration and support (24% vs 27.9% in our work) as well as personal stories (32% vs 17.0%), while the topic of education and research appeared less frequently on Instagram posts (11% vs 7.2%). Of note, content may vary due to time or certain events; Gochi et al [22] found significant changes in topics during Endometriosis Awareness Month in March when compared to February in 2020.

Regarding engagement, our results showed that post category had a substantial effect on post engagement, a correlation that had been previously observed [5]. Furthermore, we were able to display major differences in engagement between Instagram and Facebook. It is commonly known that engagement rates vary between social media platforms; content marketing providers assume “good” engagement rates in the range of 1% to 2% for Facebook, and 2% to 3% on Instagram [30].

As expected, we noticed higher user engagement on Instagram than on Facebook throughout all categories. When compared with the benchmarks described above, humoristic posts had exceptional engagement rates of more than 4% on Instagram, while many categories performed in the expected range of 2% to 3% (ie, “personal story,” “inspiration and support,” “awareness,” “other,” “education and research,” “scientific inquiries,” and “patient requests”). Instagram’s superiority in this regard has been explained as being caused by its format, as users only see one post per screen, urging them to either engage or scroll past [30]. Different mechanisms apply for Facebook. Due to the fact that posts originating from pages are broadcast to a targeted minority of followers, who are more likely to engage, it has been described that Facebook has reduced the reach of such posts over the last years [30], possibly resulting in reduced engagement. Typically, humoristic or controversial content generates strong engagement, and other authors reported strong engagement with posts about oral contraceptives, intrauterine devices, or cancer [6].

Even though medical professionals only accounted for 15% of the Facebook pages and 5% of the Instagram accounts investigated, some of these physicians were able to connect and communicate with the community, rather than acting as passive bystanders [31]. As shown in our study, posts from health care professionals were able to generate high user engagement on Facebook. Although associated with a significant investment of mostly unpaid time as well as exposure, this additional information shared by medical professionals may lead to a more balanced and evidence-based presence of information.

### Strengths

Several strengths of our study are noteworthy. Besides YouTube, which is mainly video based and was, therefore, not investigated, Facebook and Instagram are the two largest social media sites in the world, with around 50% of users being female. The approach of searching within Facebook’s and Instagram’s own search functions resembles a realistic user scenario when searching for endometriosis-related content on social media. In addition, a large number of posts that were shared with over 1 million followers were examined. By using a codebook, a profound and established method of categorizing posts was applied, and the employment of two examiners, together with an arbitrator in case of disagreement, provided for additional objectivity. Lastly, analyzing data from two social media platforms with a partially overlapping user base allowed for identification of content-related differences between both services.

### Limitations

Social media platforms are dynamic and ever-changing, limiting any attempt of mapping or categorizing their content. In this context, this study must be seen as a snapshot that provides insight but cannot adequately reflect the dynamic changes. Although we included two of the largest social media services, several other well-known social media sites, such as Twitter, LinkedIn, Snapchat, Pinterest, Reddit, and TikTok, have not been investigated. Therefore, no statement can be made about the distribution of topics on these services or on social media as a whole.

Facebook’s and Instagram’s proprietary search algorithms are noteworthy and have limited capabilities for end users. By presenting different search results depending on the user and location, certain pages (ie, accounts) that met the inclusion criteria may have been missed. On Instagram, sorting the results by the number of followers was not possible; this was addressed by screening the follower lists for further accounts with large follower numbers. On Facebook, sorting pages by the number of likes was possible at the time of data collection, a feature that has since been disabled. This allowed for identification of the largest Facebook pages down to 5000 followers.

Regarding participation bias, it is arguable that only a certain subgroup of patients with endometriosis, or people interested in endometriosis in general, were actively sharing, discussing, and following endometriosis-related content. Assuming this is the case, this study was a content analysis study that used a subset of available posts on two social media platforms on a given date; therefore, participation or nonresponse bias may influence any study’s findings in the first place, but it does not affect the veracity of this study’s results.

### Conclusions

Instagram and Facebook are being used intensively to share endometriosis-related information with a large number of users. The most common topics varied between platforms. Most posts offered inspiration or support, spread awareness about the disease, or covered personal issues. User engagement was higher on Instagram than on Facebook. Followers mostly engaged with posts with humoristic, awareness-generating, or personal content. Health care providers should be aware of the topics discussed online, as this may lead to an increased understanding of the needs and demands of digitally proficient patients with endometriosis. Future research should focus on topics that are trending as well as the scientific accuracy of social media content, possibly highlighting underlying drivers and interests of endometriosis-related content creators.

## Abbreviations

ERR: engagement rate by reach
